# British Home and Hospital for Incurables, Streatham

**Published:** 1905-07-29

**Authors:** 


### BRITISH HOME AND HOSPITAL FOR INCURABLES, STREATHAM.

A PLEASANT garden party was given on the 15th inst. in the grounds of this hospital. The entertainment was the outcome of a legacy left by Miss Trigg, a lady who was formerly an inmate of the institution, for the purpose of providing recreation and enjoyment for the patients. All those who were well enough to be moved were brought down, mostly in invalid couches or bath-chairs, to the lawn, where tea and fruit were served to them and their friends. The wards and balconies which contained the bedridden patients were also brightened by the presence of visitors and by the music which was played by a band in the grounds. Mr. H. H. Baber, executive chairman of the house committee, gave a short address of welcome to the guests, and invited them to visit the wards. Amongst those present were General Sir A. and Lady Montgomory-Moore, Sir Raymond and Lady West, Alderman Richards, Deputy-Mayor of Lambeth and Mrs. Richards, Dr. and Mrs. Smallwood, Dr. and Mrs. Stanim, Major and Mrs. Gascoigne, etc.

The Board of Management are making an earnest appeal for 200,000 shillings, both to enable them to pay off a banker's debt and to carry on their work of providing a home or pension for the helpless incurables applying for relief. Annual subscriptions are also solicited.

